# Protection of Anthocyanins by Food Matrix During Simulated Digestion: Comparative Analysis of Whole-Açaí Powder and Extracts

**DOI:** 10.3390/foods15020263

**Published:** 2026-01-11

**Authors:** Ravish Kumkum, Kathryn Aston-Mourney, Bryony A. McNeill, Leni R. Rivera

**Affiliations:** School of Medicine, Institute for Mental and Physical Health and Clinical Translation (IMPACT), Deakin University, Geelong 3220, Australia; k.ravish@deakin.edu.au (R.K.); k.astonmourney@deakin.edu.au (K.A.-M.); bryony.mcneill@deakin.edu.au (B.A.M.)

**Keywords:** anthocyanin, bioaccessibility, nutraceutical, functional foods, food matrix

## Abstract

Polyphenols, particularly anthocyanins, are associated with metabolic health benefits; however, whether anthocyanin extracts provide greater bioaccessibility than whole foods remain unclear. This study investigated the role of the food matrix in açaí berry, one of the richest natural sources of anthocyanins, by comparing polyphenol and anthocyanin bioaccessibility across freeze-dried whole fruit, crude extract, and purified extract. All samples underwent standardised INFOGEST in vitro digestion, and total polyphenol content (TPC), anthocyanins (ACN), and antioxidant activity were quantified using Folin–Ciocalteu, pH-differential, and DPPH assays, respectively. Intestinal-phase TPC % bioaccessibility was similar in whole fruit (58%) and crude extract (58%) but significantly lower in pure extract (43%). ACN bioaccessibility showed a pronounced matrix effect, with the highest retention in whole fruit (44%), followed by crude extract (32%), and the lowest retention in pure extract (12%). Antioxidant activity after intestinal digestion mirrored these patterns. Overall, these findings demonstrate that the natural açaí food matrix substantially preserves anthocyanin stability during digestion, resulting in higher bioaccessible levels than matrix-free extracts, suggesting that incorporating matrix components into anthocyanin-rich nutraceuticals may help support greater functional recovery during gastrointestinal digestion.

## 1. Introduction

Growing interest in fruit polyphenols has been driven by their antioxidant properties and potential protective effects against chronic diseases, including cardiovascular diseases [1], diabetes, and cancer [2]. Berries, among the richest sources of these polyphenols, are increasingly consumed as functional ingredients or concentrated extracts to overcome seasonal and geographical limitations [3]. However, a critical limitation affecting the efficacy of both whole fruits and supplements is the low and variable bioavailability of polyphenol. This has led to a proliferation of strategies aimed at enhancing polyphenol stability and absorption, including encapsulation, emulsification, and purification techniques [4]. However, many of these methods remove or alter the food matrix, a factor increasingly recognised as a determinant of polyphenol release, protection, and interaction during digestion [5].

The food matrix refers to the complex physical and chemical environment in which nutrients and bioactives are embedded. It has been shown to influence digestive stability, enzyme interactions, release kinetics. Ultimately, it determines bioaccessibility, which is defined as the fraction of a compound released from the food matrix and available for absorption in the gastrointestinal tract [6]. While some components of the matrix, such as dietary fibre and lipids, may protect polyphenols during digestion, others may inhibit their release or absorption [7,8]. Despite its importance, the role of the food matrix in polyphenol bioaccessibility remains poorly understood, especially in comparison to matrix-free supplements.

Açaí (Euterpe oleracea) a fruit native to the Amazon region, is notable for its exceptionally high anthocyanin content, placing it in fourth position among anthocyanin-rich fruits after blackcurrant, blueberry, and blackberry, based on data from the Phenol-Explorer database [9,10]. Interestingly, Açaí has shown greater antioxidant activity compared to most common anthocyanin-rich fruits including highbush blueberries, blackberries, cranberries, raspberries, strawberries and others [11,12]. In addition to its polyphenol profile, açaí is high in lipids (~49%, primarily oleic acid), rich in insoluble fibre, and contains a small fraction of soluble fibre [10,13]. Due to its exclusive cultivation in the Amazon biome, fresh açaí is rarely available outside Brazil. However, it is increasingly consumed globally in forms such as pulp, juice, freeze-dried powder, and more recently, as crude and pure extracts [14]. While whole fruits retain a complex matrix of fibre and other nutrients that may enhance or inhibit the release of bioactive compounds, extracts, typically produced through industrial processing, are concentrated and are devoid of the natural matrix of the fruit. Despite the increasing prevalence of berry-based supplements, there is a paucity of research directly comparing the gastrointestinal fate of polyphenols in whole foods versus their extracted counterparts, particularly with respect to anthocyanin-rich matrices [15].

While both bioaccessibility (fraction released during digestion) and bioavailability (fraction absorbed and utilised by the body) are critical for determining the health benefits of polyphenol intake, digestion represents the first and most crucial step. Understanding how polyphenols and anthocyanins withstand the gastrointestinal environment, characterised by variable pH, enzymatic activity, and matrix interactions, while also undergoing changes in antioxidant properties, is critical for predicting both their biological efficacy and the proportion available for absorption at the intestinal epithelium. Beyond bioavailability, evidence suggests that polyphenols released during digestion may also contribute to protecting the gastrointestinal tract itself against oxidative stress and carcinogenesis in the stomach, colon and rectum [16]. Against this background, the present study investigates the digestive stability and bioaccessibility of polyphenols and anthocyanins from açaí berries presented in three distinct forms: whole-fruit powder (with food matrix), crude extract (matrix-reduced), and pure extract (matrix-free). It further examines associated changes in antioxidant capacity across digestion phases. To our knowledge, this is among the first comparative studies to directly compare these three forms of anthocyanin-rich food to elucidate the influence of the food matrix on gastrointestinal fate. The findings are expected to generate valuable insights for nutritional guidance, functional-food innovation, and supplement development aimed at enhancing the delivery and efficacy of anthocyanin-rich ingredients.

## 2. Materials and Methods

### 2.1. Chemicals and Reagents

Freeze-dried açaí berry powder (250 g) was obtained from Purewellness (Southport, QLD, Australia). All analytical-grade chemicals and reagents used in the study—including ethanol, potassium chloride (KCl), potassium dihydrogen phosphate (KH_2_PO_4_), sodium bicarbonate (NaHCO_3_), sodium chloride (NaCl), magnesium chloride hexahydrate (MgCl_2_·6H_2_O), ammonium carbonate ((NH_4_)_2_CO_3_), sodium hydroxide (NaOH), hydrochloric acid (HCl), calcium chloride dihydrate (CaCl_2_·2H_2_O), sodium acetate, salivary α-amylase, Folin–Ciocalteu reagent, and 2,2-diphenyl-1-picrylhydrazyl (DPPH)—were purchased from Sigma-Aldrich (Castle Hill, NSW, Australia). Rabbit gastric extract (Cat. No. RGE15-1G) was obtained from Lipolytech (Marseille, France), and bovine bile extract (Cat. No. MED9.10) was sourced from Southern Biological (Nunawading, VIC, Australia). Solvent evaporation was performed using a Labconco SpeedVac concentrator. A LAQUAtwin Compact pH Meter, model pH 22 (Instrument Choice, Dry Creek, SA, Australia) was used to measure pH throughout the digestion process.

### 2.2. Preparation of Anthocyanin-Rich Extracts

Crude anthocyanin extracts were obtained via solvent extraction using 80% ethanol in a 1:20 (*w*/*v*) ratio [17]. Briefly, 0.5 g of freeze-dried açaí powder was extracted twice with the solvent, and the mixtures were centrifuged at 4000 rpm for 30 min (Thermo Fisher, Scoresby, VIC, Australia). The supernatants were pooled and evaporated to dryness using a Labconco SpeedVac concentrator at 40 °C overnight to yield the crude extract.

For the preparation of purified anthocyanins, the crude extract was reconstituted in 2 mL of acidified water (0.01% HCl) and subjected to solid-phase extraction using Sep-Pak Plus C18 cartridges (360 mg sorbent, 55–105 µm particle size; Waters, Dundas, NSW, Australia) [18]. The cartridges were preconditioned with 5 mL methanol followed by 5 mL acidified water. The reconstituted extract was loaded onto the cartridge, washed with 2 mL of acidified water to remove sugars and other polar impurities, and then eluted with 2 mL of 80% ethanol containing 0.1% HCl. The anthocyanin-rich eluate was concentrated to dryness using a SpeedVac at 30 °C and reconstituted in 1 mL of distilled water immediately prior to use. All three forms, freeze-dried powder, crude extract, and purified extract were used for subsequent in vitro digestion experiments.

### 2.3. In Vitro Digestion

In vitro digestion was performed using the standardised INFOGEST 2.0 static digestion protocol [19]. A uniform starting mass of 0.5 g was used across all sample formats to ensure comparability and isolate the effect of food matrix structure on digestive bioaccessibility. Freeze-dried açaí powder was dispersed in 1 mL of Milli-Q water and subjected to the oral, gastric, and intestinal phases to represent whole-fruit digestion. Crude and purified extracts, prepared from the same starting mass as described in Section 2.2, were reconstituted in 1 mL of water and digested through the gastric and intestinal phases only, mimicking consumption formats such as juice or dietary supplements, where oral digestion is typically bypassed.

For the oral phase, 1 mL of simulated salivary fluid containing α-amylase (75 U/mL) was added to the sample and incubated at 37 °C for 2 min in a shaking incubator. The pH was adjusted to 7.0 using 1 M NaOH or HCl as needed. Gastric digestion was initiated by adding simulated gastric fluid (1:1, *v*/*v*) containing pepsin (2000 U/mL) and lipase (60 U/mL) from rabbit gastric extract. The pH was adjusted to 3.0, and samples were incubated at 37 °C for 2 h. Intestinal digestion was carried out by adding simulated intestinal fluid (1:1, *v*/*v*) containing pancreatin (trypsin activity 100 U/mL) and bile salts (10 mM), adjusting the pH to 7.0, and incubating for a further 2 h at 37 °C. Following digestion, samples were centrifuged, and the supernatants were collected and stored at −80 °C for further analysis.

### 2.4. Total Polyphenol Content

Total polyphenolic content (TPC) was quantified using the Folin–Ciocalteu method, as described by Subbiah et al. [20], with minor modifications for a 96-well-plate format. Briefly, 50 µL of the digested or undigested sample supernatant (0.05 mg/mL, diluted 20-fold) was added to each well of a 96-well microplate (Costar, Corning, NY, USA), followed by 75 µL of a 1:3 (*v*/*v*) diluted Folin–Ciocalteu reagent. Then, 75 µL of 10% (*w*/*w*) sodium carbonate solution was added, and the mixture was incubated at 25 °C for 60 min in the dark. Absorbance was measured at 765 nm using a microplate reader (Bio-Rad, Thermo Fisher Scientific, Waltham, MA, USA). A standard curve was prepared using gallic acid solutions (0–300 µg/mL), with an R^2^ value of 0.98. TPC was calculated from the calibration curve and expressed as mg gallic acid equivalents (GAE) per mL of sample, normalised to the dry weight of the original material.

### 2.5. Total Anthocyanin Content

Total anthocyanin content (ACN) was measured using the pH differential method described by Lee et al. [21], employing potassium chloride buffer (0.025 M, pH 1.0) and sodium acetate buffer (0.4 M, pH 4.5). Sample supernatants from each digestion phase and undigested controls were prepared at a concentration of 0.05 mg/mL and diluted 50-fold using the corresponding buffers directly in a 96-well microplate. This dilution achieved a linear absorbance range suitable for quantification. Absorbance was measured at 520 nm and 700 nm using a UV-Vis spectrophotometer. Total ACN was then calculated according to the following formula:

$$\mathrm{ACN}\;(\mathrm{mg}\;C3G/g)\;=\;\frac{A\times MW\times DF\times V\times 10^{3}}{\varepsilon \times l\times W}$$

where A = absorbance (as calculated above), MW = molecular weight of cyanidin-3-glucoside (449.2 g/mol), DF = dilution factor (50), ε = molar absorptivity coefficient (26,900 L mol^−1^cm^−1^), l = path length (1 cm), 10^3^ = factor for conversion from grams to milligrams, V = total volume of extract (mL), and W = sample dry weight (g). The ACN was expressed as cyanidin-3-glucoside (mg/g, dry weight).

### 2.6. Antioxidant Activity

The antioxidant activity of the digested supernatants was quantified using a DPPH (2,2-diphenyl-1-picrylhydrazyl) radical scavenging assay in a 96-well microplate. A 400 µM DPPH working solution was prepared in absolute ethanol and protected from light throughout the assay. This working solution produced an absorbance of approximately 0.9–1.0 at 517 nm when measured against an ethanol blank.

Prior to analysis, digestion supernatants were diluted in ethanol to ensure measurements fell within the linear range of the assay. Diluted supernatants were then mixed with the DPPH solution at a 1:1 ratio in the microplate. Ethanol containing DPPH served as the assay control, ethanol alone as the blank, and diluted supernatants mixed with ethanol were used as colour controls. The plate was covered with foil, incubated at room temperature for 30 min in the dark, and absorbance recorded at 517 nm.

The absorbance was corrected for intrinsic colour by subtracting the corresponding colour control. Radical scavenging activity was calculated as the percentage inhibition relative to the control using following formula [22]:

$$\%\;\mathrm{Inhibition}\;\mathrm{or}\;\mathrm{Radical}\;\mathrm{Scavenging}\;\mathrm{activity}\;(\mathrm{RSA})\;=\;\frac{Acontrol-Asample}{Acontrol}\times 100$$

where Acontrol is the absorbance of the DPPH working solution without sample, and Asample is the absorbance of the DPPH solution with the sample.

### 2.7. Percentage Bioaccessibility

Percentage bioaccessibility, also known as the recovery index or percentage recovery [23], quantifies the proportion of polyphenolic and anthocyanin compounds released and available for absorption during each digestive phase relative to the undigested sample [24]. Although some studies use the term “percentage recovery,” this paper adopts “percentage bioaccessibility” to better reflect the digestion context.

$$\%Bioaccessibility=\frac{Bioaccessible\;amount\;of\;digested\;sample}{Total\;extractable\;amount}\times 100$$

### 2.8. Statistical Analysis

All experiments were performed in three independent replicates, with analytical measurements performed in triplicate, and the results are presented as mean ± standard deviation. Statistical analyses were performed using GraphPad Prism (version 9, GraphPad Software, San Diego, CA, USA). A two-way analysis of variance (ANOVA) was used to assess the effects of digestion phase (undigested, oral, gastric, intestinal) and sample type (freeze-dried powder, crude extract, purified extract) on total polyphenolic content, anthocyanin content, and antioxidant activity in açaí berry samples. Comparisons were made between digested samples, undigested controls, and enzyme blanks. Tukey’s post hoc test was used for multiple comparisons, and statistical significance was set at *p* < 0.05.

## 3. Results and Discussion

To establish a reference for bioaccessibility calculations, the TPC and ACN of the freeze-dried açaí powder were first determined using 80% acidified ethanol. This method yielded the highest recovery values (53.4 ± 0.9 mg GAE/g TPC and 14.97 ± 1.7 mg C3G/g ACN), representing the total extractable amount. These results agree with previously reported ranges for freeze-dried açaí extracted with 70% methanol (TPC: 4.3–44.7 mg GAE/g; ACN: 3.6–14.3 mg C3G/g, d.w) [25]. By comparison, extraction with acidified water, performed to simulate the maximum potential recovery under aqueous gastrointestinal conditions, yielded lower values of 34.5 ± 1.2 mg GAE/g TPC and 12.43 ± 1.9 mg C3G/g ACN. Consequently, the ethanol-extracted values were utilised as reference standard for all percentage bioaccessibility calculations.

To differentiate the factors influencing bioaccessibility, the digestion protocol for each sample (freeze-dried powder, crude and pure extract) included three parallel conditions: (i) the standard digested sample, subjected to the complete protocol containing all simulated fluids and enzymes; (ii) a ‘sham-digested’ control, in which digestive fluids were replaced with Milli-Q water to assess natural matrix release in the absence of digestive factors; and (iii) an enzyme-free blank, consisting of the sample with digestion fluids but without enzymes, to isolate the effects of pH and ionic strength distinct from enzymatic activity.

### 3.1. Total Polyphenol Content

**Figure 1:**
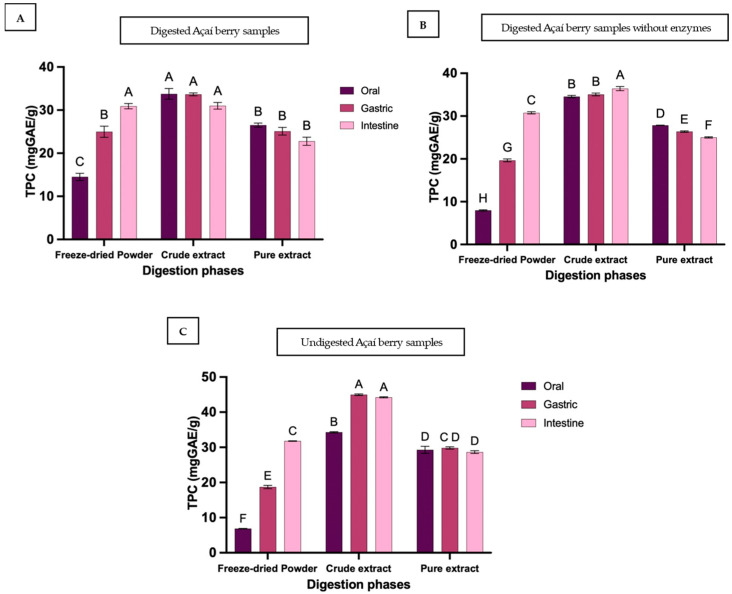
Total polyphenol content (TPC) of açaí berry samples during in vitro digestion. TPC is expressed as milligrams of gallic acid equivalents per gram dry weight (mg GAE/g) for freeze-dried powder, crude extract, and pure extract across oral, gastric, and intestinal phases. Panels show (**A**) enzymatic digestion, (**B**) digestion without enzyme, and (**C**) control or undigested samples (all digestive fluids replaced with Milli-Q water). Bars represent mean ± SD (n = 3). Different superscript letters indicate statistically significant differences among samples and phases (*p* < 0.05). For crude and pure digested extracts, the oral phase represents a sham digestion to serve as a baseline.

In the freeze-dried powder, TPC increased progressively through digestion (Figure 1), rising from 14.5 ± 1.5 mg GAE/g in the oral phase to 25.0 ± 2.2 mg GAE/g in the gastric phase, and reaching 31.0 ± 1.1 mg GAE/g in the intestinal phase, with significant increases observed at each stage (*p* < 0.0001). These amounts correspond to 27%, 47% and 58% bioaccessibility (Table 1). In contrast, the crude extract peaked in the gastric phase at 33.7 ± 0.6 mg GAE/g (63%) before declining slightly to 31.0 ± 1.3 mg GAE/g (58%) in the intestinal phase, whereas the pure extract decreased from 25.1 ± 1.5 mg GAE/g (47%) to 22.8 ± 1.6 mg GAE/g (43%). These intestinal-phase decreases in both extracts were not statistically significant. The initially high levels of TPC in both crude and pure extracts likely reflect pre-extraction of polyphenols from the food matrix during processing.

Overall, these results suggest that, unlike the freeze-dried powder, TPC in extracts may be more prone to minor losses during intestinal digestion, possibly due to the absence of protective food matrix components. Compared to undigested extracts, the intestinal bioaccessible TPC was significantly lower in both extracts, with 25% losses in crude extract and 9% losses in pure extract. From the gastric to the intestinal phase, digested extracts lost 8–9% TPC, whereas the freeze-dried powder showed a significant 24% increase between these phases, underscoring the protective effect of a complex food matrix compared to residual matrix in crude extract [15,26,27]. This divergence in stability between the complex food matrix and the extracts was statistically corroborated by a significant interaction between sample type and digestion phase (Two-way ANOVA, *p* < 0.0001).

The reduction in TPC in extracts may be attributed to the absence of protective components such as dietary fibres, leaving them more vulnerable to alkaline-induced degradation processes like oxidation, polymerization, and structural rearrangements [28,29]. In contrast, the intact cellular structure of the freeze-dried powder likely retained polyphenols that were gradually liberated during digestion. These patterns align with previous reports that purified or enriched extracts often display greater initial bioaccessibility but lower intestinal retention compared to intact fruit matrices [27]. Food matrix protection has been attributed to several mechanisms, including entrapment of polyphenols by dietary fibres and polysaccharides. This process enhances retention by increasing viscosity, reducing peristaltic mixing, and delaying enzymatic access [30]. Our findings in the freeze-dried powder showed a gradual release of polyphenols at each digestion stage. These results align with the proposal by Tagliazucchi et al. that the gastrointestinal tract itself acts as an extractor of polyphenols [31]. This does not apply to extracts, where maximum polyphenolic content is released early due to pre-extraction or absence of matrix. Consistent with our results, studies on grape and maqui berry reported that matrix-bound compounds exhibit sustained release and greater intestinal retention [30,32]. In maqui berry, adding dietary fibres during in vitro digestion increased intestinal bioaccessibility of phenolics and flavonoids by up to 22% compared to fibre-free extracts. Anthocyanins and other polyphenols were stabilised through interactions with fibres, particularly gums such as sodium carboxymethyl cellulose, xanthan gum, and guar gum, highlighting the role of fibre in protecting polyphenols during digestion [30].

#### 3.1.1. Role of Digestive Conditions

TPC bioaccessibility in freeze-dried powder increased significantly during the oral and gastric phases, reaching 27.2% and 46.8%, respectively, compared to undigested controls (12.9% and 35%) (Table 1). This indicates that digestive conditions, including pH and enzymatic activity, facilitate early release of matrix-bound polyphenols. Comparison with enzyme-blank groups (15% for oral phase, 37% for gastric phase) highlights the role of salivary amylase, gastric lipase, and pepsin in enhancing polyphenol release early in digestion. However, this enzymatic advantage diminished in the intestinal phase, where bioaccessibility was similar regardless of enzyme inclusion (58–60%), suggesting intestinal enzymes do not further increase polyphenol release. In contrast, crude and pure extracts exhibited similar gastric-phase TPC whether digested or without enzymes, indicating enzyme-mediated release is more pronounced in the presence of a food matrix. Both extracts showed significant reductions in intestinal-phase TPC, consistent with findings in black rice [33], *Kadsura coccinea* fruit [34], blackberries [35].

#### 3.1.2. Matrix-Bound Polyphenols and Colonic Fermentation

Notably, about 40% of polyphenols remained non-bioaccessible after intestinal digestion of freeze-dried powder. Of the total 53.4 mg GAE/g, only 34.5 mg GAE/g was released, suggesting a substantial fraction either escaped digestion or remained bound to the matrix. Partial retention was also noted in the crude extract where ~17% appeared entrapped within residual matrix, potentially available for later biological activity. These matrix-bound compounds may reach the colon, undergo microbial metabolism, and contribute to health benefits via colonic fermentation [34]. Thus, matrix-bound polyphenols represent an important reservoir of bioactives, offering sustained release beyond immediate bioaccessibility. Preserving the natural food matrix or using minimally processed forms may therefore optimise both stability and functional delivery along the gastrointestinal tract.

Interestingly, the baseline (undigested) TPC was markedly lower in the purified extract compared to the crude extract (Figure 1A). This reduction indicates that the solid-phase extraction process successfully removed non-anthocyanin phenolic compounds (such as phenolic acids and other flavonoids) that otherwise contribute to the total phenolic measurement in the crude sample.

### 3.2. Anthocyanin Content

**Figure 2:**
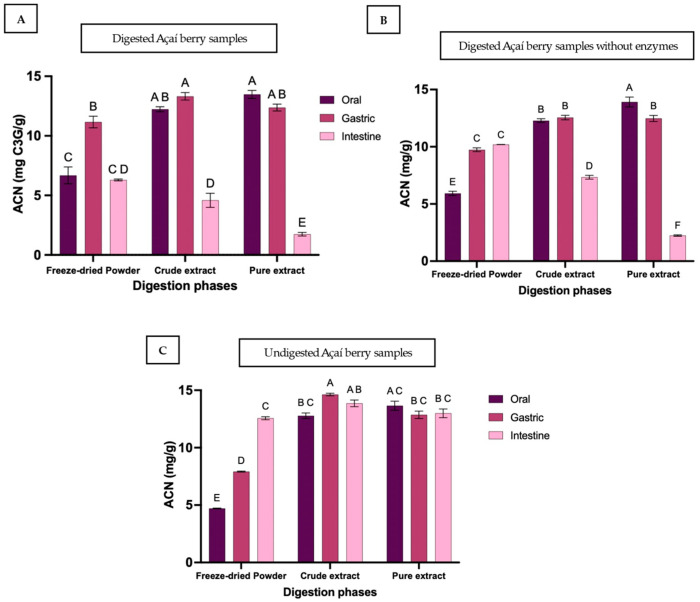
Total monomeric anthocyanin content (ACN) of açaí berry samples during in vitro digestion. ACN is expressed as milligrams cyanidin equivalents per gram dry weight (mg C3G/g) for freeze-dried powder, crude extract, and pure extract across oral, gastric, and intestinal phases. Panels show (**A**) enzymatic digestion, (**B**) digestion without enzyme, and (**C**) control or undigested samples (all digestive fluids replaced with Milli-Q water) Bars represent mean ± SD (n = 3). Different superscript letters indicate statistically significant differences among samples and phases (*p* < 0.05). For crude and pure digested extracts, the oral phase represents a sham digestion to serve as a baseline.

In the freeze-dried powder, ACN bioaccessibility increased significantly from 6.7 ± 1.2 mg C3G/g (47%) in the oral phase to 11.2 ± 0.8 mg C3G/g (78%) in the gastric phase then declined significantly to 6.3 ± 0.1 mg C3G/g (44%) in the intestinal phase, returning to levels comparable to the oral phase (*p* = 0.9985) (Figure 2 and Table 2). Similarly, ACN levels in the crude extract peaked in the gastric phase at 13.3 ± 0.5 mg C3G/g (92%) and declined significantly to 4.6 ± 1.0 mg C3G/g (32%) in the intestinal phase (*p* < 0.0001), whereas the pure extract declined more dramatically from 12.4 ± 0.5 mg C3G/g (86%) to 1.8 ± 0.3 mg C3G/g (12%) (*p* < 0.0001). Final intestinal ACN levels were similar in the freeze-dried powder and crude extract but were significantly lower in the pure extract, both compared to freeze-dried powder and crude extract, underscoring the vulnerability of anthocyanins in the absence of a protective food matrix.

These patterns demonstrate a clear food matrix effect, strongly influenced by pH. Although extracts initially contained high bioaccessible ACN, they were highly susceptible to intestinal degradation, whereas the freeze-dried powder showed a more gradual release, resulting in higher ACN retention at the end of digestion. Percentage losses during the intestinal phase were 50% for freeze-dried powder, 67% for crude extract, and 75% for pure extract, comparable to what has been previously shown for red cabbage (58% with matrix, 87% in extract) [15]. These recovery values should be interpreted in the context of the analytical method’s specificity for intact forms, as detailed in Section 3.2.2. Further, the lowest intestinal ACN aligns with the substantial ACN degradation reported in black rice (73%) [36], plum (93%) [31], grape (79%) [32], and pomegranate juice (97%) [37]. This decline is largely attributed to structural transformations that occur under intestinal conditions. Specifically, at neutral pH, the flavylium cation undergoes hydration to yield colorless chalcone and pseudobase forms, causing spectral loss [15,26,27,38]. Cyanidin-3-glucoside, the predominant anthocyanin in many fruits, is particularly prone to degradation under neutral to alkaline pH, leading to the formation of phenolic metabolites such as protocatechuic acid and phloroglucinol aldehyde [39,40]. The overall low bioavailability of anthocyanins is probably due to their pronounced instability at alkaline pH, as highlighted in multiple studies [36,37,41,42,43,44,45]. The presence of a food matrix, however, helps preserve a greater proportion of anthocyanins, leading to higher amounts available during the intestinal phase and maybe even during colonic fermentation. Overall, anthocyanins show lower stability during gastrointestinal digestion compared to TPC, consistent with reports in pomegranate [46], black rice [33] blackberries [35] and grapes [32]. Beyond these general trends, the enhanced stability observed in the whole-fruit powder can be attributed to the unique compositional profile of açaí. Unlike most other anthocyanin-rich berries, açaí is exceptionally high in lipids (>50% dry weight), composed predominantly of oleic acid resembling the lipid profile of olives [47,48]. Recent research indicates that lipids facilitate molecular interactions, such as hydrophobic effects and hydrogen bonding, which allow anthocyanins to be incorporated into the lipid phase of micelles. This encapsulation effectively shields the phenolic hydroxyl groups from degradation at neutral pH [49]. Additionally, the significant fraction of dietary fibre (25%) in the whole fruit may act as a physical barrier, limiting the diffusion of digestive enzymes and retarding the rapid chemical degradation observed in the extracts [50].

#### 3.2.1. Influence of Enzymes and pH

Comparisons between undigested, digested, and enzyme-blank groups provided insights into the influence of enzymes and pH on anthocyanin bioaccessibility. In freeze-dried powder, bioaccessible ACN levels in the oral and gastric phases were significantly higher than in undigested samples (4.7 ± 0.05 mg C3G/g, 33%; 7.9 ± 0.07 mg C3G/g, 55%; *p* < 0.001), but comparable to enzyme-blank values (6.0 ± 0.3 mg C3G/g, 41%; 9.7 ± 0.3 mg C3G/g, 68%), (Table 2) indicating that pH, rather than enzymatic activity, primarily drives anthocyanin release in the early stages of digestion [38]. In the intestinal phase, ACN bioaccessibility declined significantly in both digested (6.3 ± 0.1 mg C3G/g, 44%) and enzyme-blank groups compared to undigested samples, with levels in the enzyme-treated group significantly lower than in the enzyme-blank, suggesting a notable degrading effect of pancreatin in the presence of a food matrix, alongside high pH. For crude and pure extracts, enzyme presence did not affect ACN levels in the gastric phase, and no significant enzyme-driven losses occurred in the intestinal phase. This clearly indicates that chemical instability rather than enzymatic hydrolysis predominates in extracts. Similar findings were reported for chokeberry juice, where intestinal ACN losses were attributed mainly to chemical instability [51].

#### 3.2.2. Methodological Considerations

Anthocyanin quantification was performed using the pH differential method, which detects intact monomeric anthocyanins but not degradation products or conjugated metabolites. This limitation partly explains the apparent low recovery observed in extracts after digestion. Once anthocyanins undergo structural breakdown, particularly at the higher pH of the intestinal environment, the flavylium cation is irreversibly converted to chalcone and pseudobase forms or further degrades into phenolic acids, which lack the chromophore detected by this method. Previous studies have shown that anthocyanidins (aglycones) and chalcones are among the earliest intermediates, with chalcones reported as the most abundant metabolites under simulated intestinal conditions [38]. Stability also depends on structural factors such as glycosylation and acylation, with acylated forms generally more resistant to degradation. These differences in chemical composition explain the variation in ACN bioaccessibility between whole fruit and extracts. Therefore, the bioaccessibility values reported here represent only the fraction of intact anthocyanins preserved through digestion, while excluding metabolites that may still contribute to biological activity. Nevertheless, quantifying intact anthocyanins remains essential as a direct measure of digestive stability and the protective capacity of the food matrix.

### 3.3. Antioxidant Activity

**Figure 3:**
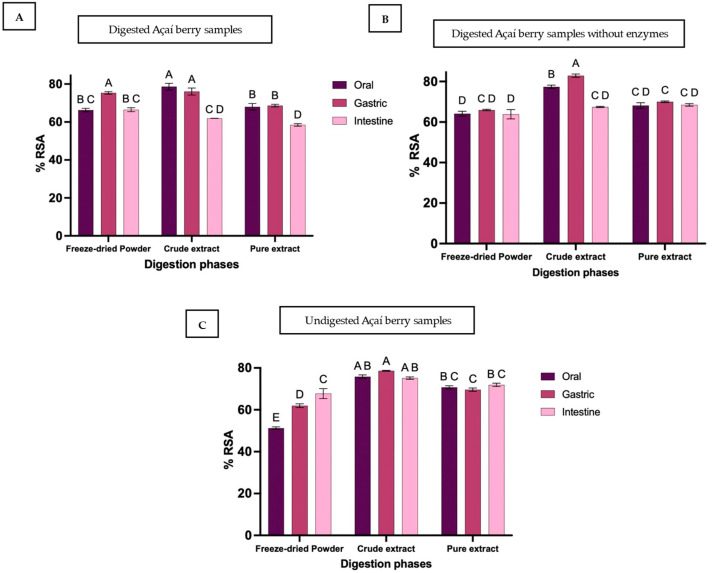
Antioxidant activity of açaí berry samples during in vitro digestion, expressed as percentage radical scavenging activity (%RSA), for freeze-dried powder, crude extract, and pure extract across oral, gastric, and intestinal phases. Panels show (**A**) enzymatic digestion, (**B**) digestion without enzyme, and (**C**) control or undigested samples (all digestive fluids replaced with Milli-Q water) Bars represent mean ± SD (n = 3). Different superscript letters indicate statistically significant differences among samples and phases (*p* < 0.05). For crude and pure digested extracts, the oral phase represents a sham digestion to serve as a baseline.

Antioxidant activity, expressed as the percentage of radical scavenging activity (%RSA) measured using the DPPH method, reflects the ability of polyphenols to donate electrons and neutralise DPPH radicals [52]. RSA varied across digestion phases and among sample groups (Figure 3), following patterns more closely aligned with ACN than with TPC.

In freeze-dried powder, %RSA increased significantly from 66.3 ± 1.6% in the oral phase to 75.3 ± 1.2% in the gastric phase (*p* < 0.0001). It subsequently declined to 66.5 ± 1.8% in the intestinal phase (*p* < 0.0001), returning to levels comparable to the oral phase. Both crude and pure extracts exhibited similar trends, with significant decreases from gastric to intestinal phase (78.6 ± 3.0% to 62.0 ± 0.04% in crude extract; 68.6 ± 1.2% to 58.5 ± 1.2% in pure extract; *p* < 0.0001). Gastric-phase RSA was not significantly different from their initial baseline (sham oral phase) for either extract, suggesting that low gastric pH enhances polyphenol release primarily when a food matrix is present, whereas extracts lack this effect.

Interestingly, enzyme-blank samples did not fully replicate these patterns. RSA remained stable across all stages for both freeze-dried powder and pure extract, with no significant differences observed between phases (Figure 3). In contrast, the crude extract displayed a significant increase in the gastric phase, followed by a significant decline in the intestinal phase. At the end of intestinal digestion, antioxidant activity in freeze-dried powder was comparable to undigested samples, whereas both extracts exhibited significant reductions (18% and 15% losses, respectively). Similar intestinal-phase losses have been reported for maqui berry (75.4%) [53], blueberry (50%) [54], and red-fleshed apples [55]. These decreases align with previous studies using ABTS, DPPH, FRAP [55], and CUPRAC assays [56], and are largely attributed to reductions in polyphenolic content, particularly anthocyanins [41,57]. Notably, these reductions in antioxidant activity were proportionally smaller than the corresponding declines in anthocyanin content. While anthocyanin bioaccessibility decreased by approximately 50–70% across digestion phases, the reduction in RSA was limited to ~15–18%, indicating that non-anthocyanin phenolics and matrix-associated compounds continue to contribute to overall antioxidant capacity. This disparity highlights the buffering effect of the food matrix and the role of residual polyphenols in maintaining antioxidant protection despite substantial anthocyanin degradation. The higher RSA observed in the crude extract compared to the purified extract likely reflects the synergistic interaction between anthocyanins and additional non-anthocyanin phenolics retained in the crude fraction. Solid-phase extraction selectively enriches anthocyanins while removing other phenolic compounds that also contribute to DPPH scavenging, resulting in a lower overall antioxidant response in the purified extract despite its higher anthocyanin specificity.

Despite these losses, the %RSA did not fall below 50% in any sample, suggesting that sufficient bioaccessible polyphenols remain throughout digestion to provide antioxidant protection [32]. This protective effect was most pronounced in freeze-dried powder and crude extract, particularly in the gastric phase, emphasizing the role of the food matrix in maintaining antioxidant potential. Variability in antioxidant activity observed here is consistent with previous reports on commercial fruit juices. A study using FRAP and DPPH assays on 25 juices found substantial differences post-digestion: orange and apple juices showed no significant change, grapefruit and pineapple decreased, while cranberry, red grape, and pomegranate increased [58]. These findings highlight that antioxidant activity depends on polyphenol type, structural characteristics, matrix composition, and processing conditions. Consequently, since antioxidant assays are selective for specific structural forms, their results should be interpreted with caution [23,52]. This reinforces the need to consider both matrix effects and digestion conditions when evaluating antioxidant activity, as demonstrated in the present study with açaí freeze-dried powder and extracts.

### 3.4. Limitations and Future Directions

This study provides a comprehensive comparison of how different food matrix structures from whole fruit to purified extracts influence the digestive fate of anthocyanins. However, certain methodological constraints should be considered. While freeze-dried powder was selected for its convenience and extended shelf life, it may not fully replicate the digestion behaviour of intact fruit. For example, in grapes, polyphenols are primarily released from the pulp during the oral phase and from the skin during the gastric phase [32]. While freeze-dried powder contains both skin and pulp, structural disruption during processing may alter release dynamics [59], potentially overestimating intestinal retention due to disrupted cellular mechanics. Additionally, phenolic compounds can form complexes with macromolecules such as proteins, starch, and dietary fibres, reducing their bioaccessibility [38]. These interactions may be modified by freeze-drying, warranting further investigation to determine whether significant differences exist between freeze-dried and fresh fruit during digestion.

While this study focused on the bioaccessible fraction of anthocyanins, it is important to consider that anthocyanins, especially cyanidin derivatives, are extensively metabolised during digestion [60]. They undergo transformations such as deglycosylation, decarboxylation, and cleavage, forming smaller phenolic metabolites like catechuic, caffeic, gallic, vanillic, and p-coumaric acids many of which are also considered bioactive [35]. Consequently, measuring only parent anthocyanins may underestimate true bioavailability and biological impact [61]. In addition, the food matrix can influence the release and absorption kinetics of these compounds, as shown in human studies where polyphenol-fibre interactions delayed plasma appearance. Future studies should therefore aim to quantify both parent compounds and key metabolites to provide a comprehensive picture of anthocyanin bioavailability using techniques such as HPLC-MS [62]. Further integration of dynamic gastrointestinal digestion and colonic fermentation models, coupled with advanced metabolite profiling approaches (e.g., UPLC-HRMS), would enable improved characterisation of post-intestinal transformation and microbial-derived metabolites. Lastly, future work would benefit from exploring polyphenol interactions with specific nutrients, particularly polysaccharides and proteins, given their widespread use as nanocarriers in encapsulation technologies [63].

### 3.5. Recommendations

These findings reinforce the nutritional advantage of consuming whole fruits over isolated extracts. The natural food matrix not only provides structural protection to anthocyanins during digestion but also promotes gradual and sustained release throughout the gastrointestinal tract. When fresh berries are not available, minimally processed forms such as freeze-dried powders may offer higher anthocyanin bioaccessibility compared to purified extracts.

From a supplement and nutraceutical perspective, the marked degradation of anthocyanins in pure extracts highlights the need for advanced delivery strategies to preserve their integrity and functionality. Techniques such as encapsulation, enteric coatings, and embedding bioactives within protective matrices could enhance anthocyanin stability and improve targeted intestinal release. Aligning release profiles with the dynamic digestive environment may further optimise bioefficacy. Notably, while this study used a consistent dose of 0.5 g for all forms of açaí, commercial supplements often contain extracts concentrated 4 to 50 times higher. These findings can inform rational dose selection and guide the design of polyphenol-rich products with improved bioaccessibility.

Overall, this work highlights that processing methods and matrix integrity profoundly influence the digestive stability and bioaccessibility of anthocyanin-critical factors for dietary guidance and functional-food development.

## 4. Conclusions

This study highlights the critical role of the food matrix in shaping the digestive fate of bioactive compounds. While processing into extracts improves immediate release, it compromises digestive stability and removes the protective and sustained-release properties provided by the whole food matrix. In contrast, whole fruit retains structural components that support gradual polyphenol release and potential colonic delivery, which may extend bioactivity beyond the small intestine. Anthocyanin behaviour appears particularly sensitive to chemical conditions, especially pH, alongside matrix effects. These findings emphasise that bioaccessibility is not solely about quantity; the form, timing, and pattern of release are equally critical. The fraction available at the end of digestion directly influences absorption, cellular uptake, and microbial metabolism, all of which underpin health benefits. Understanding matrix–compound interactions is therefore essential for guiding dietary strategies and designing effective supplement delivery systems. Ultimately, processing and structural modifications exert profound effects on digestive stability and bioavailability and should be carefully considered in both nutritional research and functional-food development.

## Figures and Tables

**Table 1:** Percentage bioaccessibility of Total Polyphenol content in different açaí berry samples during in vitro digestion.

Sample Type	Phase	Digested (%)	Enzyme Blank (%)	Undigested (%)
Freeze-dried powder	Oral	27.17 *^c^*	14.92 *^h^*	12.87 *^f^*
	Gastric	46.78 *^b^*	36.81 *^c^*	35.04 *^e^*
	Intestinal	57.9 *^a^*	57.61 *^c^*	59.55 *^c^*
Crude Extract	Sham oral	63.24 *^a^*	64.75 *^b^*	64.23 *^b^*
	Gastric	63.03 *^a^*	65.67 *^b^*	84.23 *^a^*
	Intestinal	58.06 *^a^*	68.22 *^a^*	82.84 *^a^*
Pure Extract	Sham oral	52.17 *^b^*	52.17 *^d^*	54.87 *^d^*
	Gastric	47.02 *^b^*	49.42 *^e^*	55.84 *^cd^*
	Intestinal	42.66 *^b^*	46.85 *^f^*	56.64 *^d^*

Bioaccessibility (%) was calculated relative to the total extractable amount determined by 80% acidified ethanol (14.97 ± 1.7 mg C3G/g for freeze-dried powder). Different superscript letters within the same column indicate significant differences between digestion phases and sample types (*p* < 0.05).

**Table 2:** Percentage bioaccessibility of Anthocyanins in different açaí berry samples during in vitro digestion.

Sample Type	Phase	Digested (%)	Enzyme Blank (%)	Undigested (%)
Freeze-dried powder	Oral	46.36 *^c^*	41.07 *^e^*	32.68 *^e^*
	Gastric	77.5 *^b^*	67.6 *^c^*	54.93 *^d^*
	Intestinal	43.7 *^cd^*	70.76 *^c^*	87.18 *^c^*
Crude Extract	Sham oral	84.9 *^ab^*	85.24 *^b^*	88.72 *^bc^*
	Gastric	92.42 *^a^*	87.13 *^b^*	101.49 *^a^*
	Intestinal	31.85 *^d^*	50.93 *^d^*	96.19 *^ab^*
Pure Extract	Sham oral	93.55 *^a^*	96.58 *^a^*	94.77 *^ac^*
	Gastric	85.89 *^ab^*	86.58 *^b^*	89.24 *^bc^*
	Intestinal	12.13 *^e^*	15.52 *^f^*	90.14 *^bc^*

Bioaccessibility (%) was calculated relative to the total extractable amount determined by 80% acidified ethanol (14.97 ± 1.7 mg C3G/g for freeze-dried powder). Different superscript letters within the same column indicate significant differences between digestion phases and sample types (*p* < 0.05).

## Data Availability

The original contributions presented in this study are included in the article. Further inquiries can be directed to the corresponding author.
